# Supplementary material for: Opening decision spaces: A case study on the opportunities and constraints in the public health sector of Mpumalanga Province, South Africa
Source: PLoS One. 2024 Jul 5;19(7):e0304775. doi: 10.1371/journal.pone.0304775 (PMC11226100; doi:10.1371/journal.pone.0304775)
Supplement: Attachment — Submitted filename: response to reviewers PLOS One.docx [file pone.0304775.s002.docx]

Many thanks for your very constructive review. Please find our responses in blue below.

Reviewer #1: Opening decision spaces: a case study on the opportunities and constraints in the public health sector of Mpumalanga Province, South Africa

General comments
This is a well written paper on a very important issue for federating low and middle income countries where the subnational level are mandated to implement health policies. Below are specific comments and suggestions for minor revisions

Specific comments
Abstract
Line 25..remove the, and leave as ....three core domains

This has been done.

Key words... I would have expected to see ‘ Decentralisation’ as a key word, since it’s the principle at the core of subnational decision making

Thanks for noting this omission, this was related to the fact that the health system was decentralised some time ago in South Africa (so this is not an ongoing process, as it is in many settings where studies are conducted). However, we agree that it is relevant here and have now added it.

Introduction
I suggest that it may be necessary to further explain ( in a few sentences), the model of decentralization ( deconcentration, devolution etc.) that is in practice as this may have resonance for the findings. And sometimes actors, in practice, deviate from the model prescribed by their constitution, especially in LMICs

This is an administrative decentralisation (not decongestion or devolution), with functions mandated to the provincial level in particular. This is explained in the first paragraph but we have added this qualifier. Later in the paper some of the challenges of resource flows still emanating from the central level are elaborated.

Methods
I commend the detailed writing in this section and suggest a few revisions, as follows:
Lines 160-165
It will be good to rank the socio-economic status of Mpumalaga among the other provinces, for the wider readership to fully appreciate the context, especially as Capacity ( resource... financial etc.) is one of your key study domains. The impact of resource capacity on the decision space may well differ in a better resourced provice, like the Western Cape, for instance.

We have added a comment on the GDP per capita, though note that there are equalisation grants so resources for health care are not solely dependent on the local economy.

Line 195....... remove one ‘from’

Done

Line 202...Edit sentence

Done, thank you for picking up this typo.

Line 211..... Though referenced, I think a sentence on the purpose of selecting these particular three villages can be included here, without the reader going to seek out the Reference.

We have added a short description on how these villages were selected.

Results
Findings have also been reported in a detailed manner
Given the long study period, (2015-2020), albeit retrospective, were there fluctuations observed in the decision spaces at the various levels and what factors (political, economic, change in actors) may have accounted for these?

We did not analyse trends over the period formally, and so cannot add this to findings but we have added a comment in the discussion that these features appear to remain valid, i.e. have not significantly fluctuated over the period, to our knowledge.

Discussion
You now bring in Decentralisation literature here, which is apt, but not adequately introduced at the beginning.
I also wonder if some street level bureaucracy literature can be used to explain the facility staff coping mechanism (positive or negative).

Thank you, that is indeed a relevant concept to draw on and we have added this to the discussion, though we were mindful of not adding too much length to what is already a long paper.

ACADEMIC EDITOR:

The authors are to be congratulated for this pertinent body of work. However, there are a few minor changes that will need to be addressed.

In the abstract, Line 18. Locally informed and locally relevant. Locally doesn't need to be repeated.

Deleted, thanks

Abstract results. Although capacity exists. Capacity for what. Add capacity for decision making.

Added, thanks

Introduction, line 61. VAPAR is mentioned for the first time. Put a reference.

We have added the programme website as a reference here

Data sources, line 200. Spell out VA and PAR before abbreviating. Go through the entire manuscript and fully spell out all abbreviations used for the first time e.g. Line 226 NGOs.

We have checked these and added/corrected, also to the abbreviation list

Study limitations should come after the discussion.

We have moved this sub-section down

Footnotes are not permitted. If your manuscript contains footnotes, move the information into the main text or the reference list, depending on the content.

We have turned the footnotes into references

Provide full references for references 2, 9, 17, 19, 56 and 57

We have added details to these references as required
